# Supplementary material for: Hepatitis B virus compartmentalization and single-cell differentiation in hepatocellular carcinoma
Source: Life Sci Alliance. 2021 Jul 21;4(9):e202101036. doi: 10.26508/lsa.202101036 (PMC8321681; doi:10.26508/lsa.202101036)
Supplement: Supplementary file 1 [file LSA-2021-01036_TableS1.docx]

**Supplementary Table 1.** Patient’s clinical and laboratory characteristics.

|  | **Reference range*** | **Patient P1** | **Patient P2** |
| --- | --- | --- | --- |
| **Clinical characteristics and viral status** |  |  |  |
| Age | NA | 61 | 28 |
| Sex | NA | Male | Male |
| Ethnicity | NA | Caucasian | African |
| HFE mutation C28Y/H63D/S65C | Negative | Negative | NA |
| HCC TNM | NA | T2N0M0 | T4N0M0 |
| HCC grading | NA | G1-G2 | G2 |
| Vascular invasion | NA | No | Yes |
| Tumor recurrence | NA | No | Yes |
| Systemic therapy | NA | No | Progression on Sorafenib |
| Metavir score | NA | F2 | F2 |
| HBsAg (UI/ml) | Negative | **162.96** | **4992.6** |
| Anti-HBc | Negative | **Positive** | **Positive** |
| HBeAg | Negative | Negative | **Weakly reactive** |
| Anti-HBe | Negative | **Positive** | **Positive** |
| Anti-HCV | Negative | Negative | Negative |
| Anti-HIV | Negative | Negative | Negative |
| Anti-HDV | Negative | Negative | Negative |
|  |  |  |  |
| **Perioperative laboratory tests** |  |  |  |
| Glucose (mg/dl) | 74-100 | **170** | 83 |
| Urea (mmol/l) | 2.5-7.0 | 7 | 5.01 |
| Creatinine (µmol/l) | 53.0-97.0 | 57.3 | **101.7** |
| Albumin (g/dl) | 3.5-5.0 | 4.4 | 4.1 |
| Total bilirubin (µmol/l) | 1.7-21.0 | 8.6 | 11 |
| ALT (UI/l) | 15-33 | 27 | **99** |
| AST (UI/l) | 16-32 | 21 | **261** |
| ALP (UI/l) | 41-117 | 103 | **466** |
| Gamma-gt (UI/l) | 11-69 | 43 | **752** |
| Total Cholesterol (mg/dl) | < 200 | 135 | 109 |
| Triglycerides (mg/dl) | < 150 | 101 | 42 |
| Ferritin (µg/l) | 58-319 | **445** | **728** |
| Transferrin saturation (%) | 20-40 | **13** | 25.7 |
| Hemoglobin (g/dl) | 13.0-18.0 | 12.6 | 14.5 |
| Platelet count (10^9/l) | 150-400 | 202 | 158 |
| White cell count (10^9/l) | 4.10-10.50 | 5.37 | **3.09** |
| INR | < 1.3 | 1.12 | 1.0 |
| CEA (kU/l) | < 5.0 | < 1.0 | 2.0 |
| Ca 19-9 (µg/l) | < 37.0 | 9.5 | <1.0 |
| AFP (µg/l) | < 13.0 | 2.1 | **66** |
| Total PSA (µg/l) | < 3.50 | 0.15 | NA |
| HBV-DNA (log_10_ UI/ml) | Negative | **1.85** | **2.17** |
| HBV treatment | NA | No | Tenofovir |

AFP: alpha-fetoprotein, ALP: alkaline phosphatase, ALT: alanine aminotransferase, AST: aspartate aminotransferase, CEA: carcinoembryonic antigen, HBV: hepatitis B virus, HBsAg: hepatitis B surface antigen, HCV: hepatitis C virus, HDV: hepatitis delta virus, HIV: human immunodeficiency virus, INR: international normalized ratio, PSA: prostate specific antigen. In bold are reported the values not included in the corresponding reference range. *Reference values are affected by many variables. The ranges used at the Strasbourg University Hospitals are for non-pregnant adults who do not have medical conditions affecting the results.
