# Supplementary material for: Hepatitis B virus compartmentalization and single-cell differentiation in hepatocellular carcinoma
Source: Life Sci Alliance. 2021 Jul 21;4(9):e202101036. doi: 10.26508/lsa.202101036 (PMC8321681; doi:10.26508/lsa.202101036)
Supplement: Supplementary file 2 [file LSA-2021-01036_TableS2.docx]

**Supplementary Table 2.** Respective number of cells sequenced in P1 and P2.

| **Patient** | **HCC cells** | **Lymphocytes** | **CAFs** | **TAMs** | **Endoth. cells** |
| --- | --- | --- | --- | --- | --- |
| **P1** | 142 | 2 | 262 | 80 | 0 |
| **P2** | 278 | 94 | 38 | 41 | 1 |
| **Both (=sum)** | 420 | 96 | 300 | 121 | 1 |
| **Both (%)** | 45 % | 10 % | 32 % | 13 % | 0 % |
