# Supplementary material for: Hepatitis B virus compartmentalization and single-cell differentiation in hepatocellular carcinoma
Source: Life Sci Alliance. 2021 Jul 21;4(9):e202101036. doi: 10.26508/lsa.202101036 (PMC8321681; doi:10.26508/lsa.202101036)
Supplement: Supplementary file 3 [file LSA-2021-01036_TableS3.docx]

**Supplementary Table 3.** Top-25 enriched gene sets in module 9 (containing HBV-RNA) as shown in Fig. 2E. The enrichment analysis was performed including only genes showing a similar expression profile according to a self-organizing maps analysis in hepatocytes and HCC cells. FDR: false discovery rate.

| **Gene Set Name** | **Genes** | **Overlap** | **FDR** |
| --- | --- | --- | --- |
| HSIAO_LIVER_SPECIFIC_GENES | 249 | 16 | 1.43E-12 |
| HSIAO_HOUSEKEEPING_GENES | 399 | 18 | 4.35E-12 |
| ACEVEDO_NORMAL_TISSUE_ADJACENT_TO_LIVER_TUMOR_DN | 354 | 16 | 1.15E-10 |
| STARK_PREFRONTAL_CORTEX_22Q11_DELETION_DN | 523 | 18 | 2.23E-10 |
| LEI_MYB_TARGETS | 323 | 15 | 2.98E-10 |
| WONG_MITOCHONDRIA_GENE_MODULE | 217 | 13 | 4.15E-10 |
| MOOTHA_MITOCHONDRIA | 449 | 16 | 1.84E-9 |
| WANG_TUMOR_INVASIVENESS_UP | 383 | 15 | 2.14E-9 |
| LANDIS_ERBB2_BREAST_TUMORS_324_DN | 151 | 11 | 2.22E-9 |
| HOSHIDA_LIVER_CANCER_SUBCLASS_S3 | 266 | 13 | 3.28E-9 |
| REACTOME_BIOLOGICAL_OXIDATIONS | 221 | 12 | 5.9E-9 |
| MOOTHA_HUMAN_MITODB_6_2002 | 430 | 15 | 7.34E-9 |
| KEGG_OXIDATIVE_PHOSPHORYLATION | 131 | 10 | 9.55E-9 |
| FLECHNER_BIOPSY_KIDNEY_TRANSPLANT_REJECTED_VS_OK_DN | 553 | 16 | 2.04E-8 |
| SPIELMAN_LYMPHOBLAST_EUROPEAN_VS_ASIAN_UP | 484 | 15 | 3.07E-8 |
| KEGG_METABOLISM_OF_XENOBIOTICS_BY_CYTOCHROME_P450 | 70 | 8 | 3.14E-8 |
| KINSEY_TARGETS_OF_EWSR1_FLII_FUSION_DN | 333 | 13 | 3.16E-8 |
| REACTOME_RESPIRATORY_ELECTRON_TRANSPORT_ATP_ SYNTHESIS_BY_CHEMIOSMOTIC_COUPLING_AND_HEAT_ PRODUCTION_BY_UNCOUPLING_PROTEINS | 124 | 9 | 1.06E-7 |
| ACEVEDO_LIVER_CANCER_DN | 540 | 15 | 1.06E-7 |
| LOPEZ_MBD_TARGETS | 952 | 19 | 1.06E-7 |
| REACTOME_METABOLISM_OF_AMINO_ACIDS_AND_DERIVATIVES | 374 | 13 | 1.06E-7 |
| CHIANG_LIVER_CANCER_SUBCLASS_PROLIFERATION_DN | 178 | 10 | 1.17E-7 |
| KEGG_PARKINSONS_DISEASE | 128 | 9 | 1.17E-7 |
| KEGG_HUNTINGTONS_DISEASE | 180 | 10 | 1.19E-7 |
| MOOTHA_VOXPHOS | 87 | 8 | 1.19E-7 |
