# Supplementary material for: Hepatitis B virus compartmentalization and single-cell differentiation in hepatocellular carcinoma
Source: Life Sci Alliance. 2021 Jul 21;4(9):e202101036. doi: 10.26508/lsa.202101036 (PMC8321681; doi:10.26508/lsa.202101036)
Supplement: Supplementary file 4 [file LSA-2021-01036_TableS4.docx]

**Supplementary Table 4.** Mean host factor expression in hepatocytes, HCC P1, HCC P2.

| **HOST FACTOR** | **HEPATOCYTES** | **HCC P1** | **HCC P2** | **Effect** | **p-value** |
| --- | --- | --- | --- | --- | --- |
| FOS | 2.133248 | 0.465051 | 0.224935 | E | 2.06E-58 |
| JUN | 1.805785 | 0.444527 | 0.240485 | E | 1.66E-50 |
| CEBPA | 0.100282 | 0.171114 | 0.309743 | E | 3.08E-37 |
| HNF4A | 0.198938 | 0.299371 | 0.583444 | E | 2.69E-34 |
| RXRA | 0.151461 | 0.257526 | 0.406759 | E | 1.41E-30 |
| NR0B2 | 0.261331 | 0.101959 | 0.316094 | I | 1.30E-22 |
| HLF | 0.373032 | 0.546687 | 0.223557 | E | 1.41E-16 |
| NF1 | 0.140724 | 0.259086 | 0.21921 | E | 1.16E-13 |
| PPARA | 0.247621 | 0.367407 | 0.420656 | E | 2.69E-13 |
| ZHX2 | 0.116767 | 0.112393 | 0.154149 | I | 5.12E-13 |
| POU2F1 | 0.175563 | 0.145441 | 0.19984 | E | 2.19E-10 |
| PRMT5 | 0.1 | 0.136491 | 0.128509 | I | 1.27E-09 |
| DHX9 | 0.165215 | 0.201834 | 0.235212 | E | 2.20E-08 |
| SP1 | 0.137008 | 0.151289 | 0.197504 | E | 5.08E-08 |
| STAT1 | 0.17485 | 0.214355 | 0.289109 | I | 1.22E-07 |
| PROX1 | 0.610439 | 0.508615 | 0.758761 | I | 2.64E-07 |
| HOXA10 | 0.1 | 0.100164 | 0.110828 | I | 1.48E-06 |
| SETDB1 | 0.131998 | 0.137534 | 0.167823 | I | 1.75E-06 |
| KDM1A | 0.107436 | 0.126932 | 0.135517 | E | 2.11E-06 |
| KLF15 | 0.422954 | 0.595539 | 0.492724 | E | 2.99E-06 |
| HDAC1 | 0.134379 | 0.143179 | 0.164838 | I | 4.30E-06 |
| NFIL3 | 0.269437 | 0.143501 | 0.164182 | I | 7.75E-06 |
| TBP | 0.109256 | 0.11832 | 0.123679 | E | 1.45E-05 |
| FOXA1 | 0.131688 | 0.115969 | 0.149272 | E | 1.57E-05 |
| NFYA | 0.110031 | 0.108461 | 0.1258 | E | 4.28E-05 |
| TP53 | 0.111138 | 0.133595 | 0.138572 | I | 5.64E-05 |
| NR1H4 | 0.182521 | 0.130267 | 0.176317 | E | 5.97E-05 |
| APOBEC3A | 0.125445 | 0.100286 | 0.103076 | I | 6.96E-05 |
| NFYB | 0.132188 | 0.162615 | 0.134051 | E | 0.000109 |
| SIRT3 | 0.116279 | 0.1597 | 0.126619 | I | 0.000118 |
| PRMT1 | 0.118989 | 0.145042 | 0.137654 | I | 0.000177 |
| ARNT | 0.124152 | 0.160001 | 0.149894 | I | 0.000198 |
| EZH2 | 0.105849 | 0.120241 | 0.118467 | I | 0.00027 |
| HNF1A | 0.104907 | 0.113237 | 0.11648 | E | 0.000314 |
| HIF1A | 0.167761 | 0.112819 | 0.113702 | I | 0.000641 |
| FOXA2 | 0.223462 | 0.169715 | 0.229505 | E | 0.000648 |
| CREB1 | 0.11516 | 0.141096 | 0.129999 | E | 0.001362 |
| NFKB1 | 0.124209 | 0.14349 | 0.137358 | I | 0.001682 |
| NRF1 | 0.106419 | 0.107887 | 0.12492 | E | 0.001736 |
| NFYC | 0.13724 | 0.134643 | 0.15581 | E | 0.001771 |
| STAT3 | 0.495726 | 0.573733 | 0.461245 | E | 0.002509 |
| HIVEP2 | 0.11259 | 0.11082 | 0.11581 | I | 0.01069 |
| NR2F2 | 0.201553 | 0.159725 | 0.21982 | I | 0.013228 |
| ONECUT1 | 0.114746 | 0.121583 | 0.126159 | I | 0.014514 |
| CRTC1 | 0.109008 | 0.114825 | 0.110917 | E | 0.030243 |
| ATF2 | 0.157077 | 0.139052 | 0.16172 | I | 0.031079 |
| NR5A2 | 0.170762 | 0.13703 | 0.164736 | E | 0.039788 |
| RFX1 | 0.142125 | 0.11934 | 0.151348 | I | 0.052006 |
| DDX3X | 0.335723 | 0.229423 | 0.262038 | I | 0.069224 |
| YY1 | 0.274042 | 0.185946 | 0.203309 | I | 0.069307 |
| FOXA3 | 0.133454 | 0.121519 | 0.134392 | E | 0.112485 |
| NR2F1 | 0.132995 | 0.1 | 0.104071 | I | 0.136424 |
| CREBBP | 0.193684 | 0.165874 | 0.184021 | E | 0.154405 |
| ZEB2 | 0.140737 | 0.118578 | 0.102995 | I | 0.191579 |
| SETD1A | 0.121197 | 0.11861 | 0.125619 | E | 0.192387 |
| SMC6 | 0.180806 | 0.158302 | 0.137156 | I | 0.329202 |
| KAT2B | 0.132285 | 0.114079 | 0.127441 | E | 0.435685 |
| SIRT1 | 0.149446 | 0.124324 | 0.13071 | I | 0.496234 |
| APOBEC3B | 0.1 | 0.100167 | 0.100367 | I | 0.539096 |
| SMC5 | 0.205883 | 0.172226 | 0.170195 | I | 0.742605 |

E= Enhancer, I= Inhibitor. P-value was calculated by Kruskal-Wallis test.
