# Supplementary material for: Hepatitis B virus compartmentalization and single-cell differentiation in hepatocellular carcinoma
Source: Life Sci Alliance. 2021 Jul 21;4(9):e202101036. doi: 10.26508/lsa.202101036 (PMC8321681; doi:10.26508/lsa.202101036)
Supplement: Supplementary file 5 [file LSA-2021-01036_TableS5.docx]

**Supplementary Table 5.** Top 25 marker genes prognostic for poor patients’ outcome for HCC clusters 2-5. Prognostic data and p-value are retrieved from the Human Protein Atlas project (<https://www.proteinatlas.org/>). *p*-values adjusted according to the Benjamini-Hochberg multiple testing procedure.

| **Gene** | **Marker for cluster** | **Mean cluster expression** | **Mean other clusters expression** | **Fold changes** | **p-value** | **Adjusted p-value** | **Prognostic unfavourable p-value** |
| --- | --- | --- | --- | --- | --- | --- | --- |
| CAD | 4 | 26.58851837 | 5.127279811 | 5.185697 | 8.25E-06 | 0.000166 | 1.72E-11 |
| DDX55 | 4 | 11.14703324 | 2.692621312 | 4.139844 | 0.001834 | 0.011775 | 2.11E-09 |
| NAP1L1 | 5 | 74.3179927 | 20.20094721 | 3.678936 | 0.00016 | 0.005225 | 3.29E-09 |
| POLR1A | 4 | 13.63537884 | 3.275517203 | 4.162817 | 0.00101 | 0.00737 | 3.70E-09 |
| EIF3B | 4 | 22.89381986 | 6.088485552 | 3.760183 | 0.000403 | 0.003623 | 4.41E-09 |
| RBM45 | 5 | 16.51667911 | 3.894536378 | 4.240987 | 0.003176 | 0.042052 | 5.74E-09 |
| RRAGC | 4 | 7.280712744 | 1.795871397 | 4.054139 | 0.005667 | 0.027983 | 9.97E-09 |
| GTF3C2 | 4 | 14.22974637 | 4.488719946 | 3.170112 | 0.005608 | 0.027775 | 1.21E-08 |
| LPCAT1 | 4 | 5.782692925 | 1.163766409 | 4.968946 | 0.003317 | 0.018555 | 1.88E-08 |
| EIF2B5 | 4 | 15.08196541 | 3.361132193 | 4.487168 | 0.000405 | 0.003638 | 3.38E-08 |
| SRSF2 | 4 | 106.0366953 | 34.57828994 | 3.066569 | 5.95E-06 | 0.000128 | 3.53E-08 |
| SAE1 | 5 | 45.81213578 | 10.85338183 | 4.221001 | 0.000214 | 0.006593 | 5.49E-08 |
| ILF2 | 5 | 65.8496077 | 22.08563264 | 2.981559 | 0.00188 | 0.029519 | 5.71E-08 |
| STRN4 | 4 | 7.723332063 | 0.860274483 | 8.977753 | 4.22E-05 | 0.000607 | 6.06E-08 |
| USP48 | 4 | 15.51953457 | 3.104438958 | 4.999143 | 0.000147 | 0.001619 | 6.09E-08 |
| TRPC4AP | 4 | 23.2464742 | 4.970115726 | 4.67725 | 4.99E-05 | 0.000697 | 6.25E-08 |
| WDR75 | 4 | 14.20557927 | 4.668375565 | 3.042938 | 0.007327 | 0.034122 | 6.50E-08 |
| ABCC5 | 4 | 9.016018696 | 1.448254609 | 6.225438 | 0.000257 | 0.002496 | 6.78E-08 |
| UBE2O | 4 | 17.81683109 | 2.182034239 | 8.165239 | 7.60E-07 | 2.38E-05 | 6.84E-08 |
| ZNF207 | 4 | 110.6220491 | 54.85316857 | 2.016694 | 0.003073 | 0.01757 | 7.30E-08 |
| ZBTB40 | 4 | 23.45103718 | 3.064777956 | 7.65179 | 2.25E-07 | 8.67E-06 | 7.78E-08 |
| ME2 | 5 | 7.965831276 | 0.985332257 | 8.084411 | 0.000546 | 0.012968 | 8.29E-08 |
| CAPRIN1 | 2 | 52.4614955 | 16.13637872 | 3.251132 | 0.000139 | 0.017937 | 8.67E-08 |
| SPATS2 | 4 | 7.236750897 | 1.621063278 | 4.4642 | 0.003131 | 0.017818 | 9.57E-08 |
| NAA35 | 4 | 10.49288787 | 2.808474441 | 3.736152 | 0.003968 | 0.021238 | 1.16E-07 |
