# Supplementary material for: Hepatitis B virus compartmentalization and single-cell differentiation in hepatocellular carcinoma
Source: Life Sci Alliance. 2021 Jul 21;4(9):e202101036. doi: 10.26508/lsa.202101036 (PMC8321681; doi:10.26508/lsa.202101036)
Supplement: Supplementary file 6 [file LSA-2021-01036_TableS6.docx]

**Supplementary Table 6.** Top-25 enriched gene sets in module 19 (containing HBV-RNA) as shown in Fig. 4D. The enrichment analysis was performed including only genes showing a similar expression profile according to a self-organizing map analysis in P1 cancer cells. FDR: false discovery rate.

| **Gene Set Name** | **Genes** | **Overlap** | **FDR** |
| --- | --- | --- | --- |
| LEE_BMP2_TARGETS_UP | 771 | 31 | 6.74E-8 |
| ACEVEDO_LIVER_TUMOR_VS_NORMAL_ADJACENT_TISSUE_UP | 863 | 32 | 1.24E-7 |
| HOSHIDA_LIVER_CANCER_SUBCLASS_S3 | 266 | 18 | 1.54E-7 |
| LEE_LIVER_CANCER_SURVIVAL_UP | 184 | 15 | 3.33E-7 |
| HSIAO_LIVER_SPECIFIC_GENES | 249 | 16 | 2.22E-6 |
| ACEVEDO_LIVER_CANCER_UP | 972 | 30 | 1.14E-5 |
| DIAZ_CHRONIC_MEYLOGENOUS_LEUKEMIA_UP | 1399 | 37 | 1.14E-5 |
| NUYTTEN_NIPP1_TARGETS_UP | 765 | 26 | 1.28E-5 |
| JOHNSTONE_PARVB_TARGETS_3_DN | 867 | 27 | 3.6E-5 |
| KRIGE_RESPONSE_TO_TOSEDOSTAT_6HR_DN | 940 | 28 | 4.59E-5 |
| CHIANG_LIVER_CANCER_SUBCLASS_PROLIFERATION_DN | 178 | 12 | 5.85E-5 |
| SWEET_LUNG_CANCER_KRAS_UP | 476 | 19 | 5.85E-5 |
| BRUINS_UVC_RESPONSE_LATE | 1089 | 30 | 5.85E-5 |
| KEGG_PENTOSE_PHOSPHATE_PATHWAY | 27 | 6 | 5.85E-5 |
| KRIGE_RESPONSE_TO_TOSEDOSTAT_24HR_DN | 1033 | 29 | 5.85E-5 |
| SANCHEZ_MDM2_TARGETS | 15 | 5 | 5.85E-5 |
| SMID_BREAST_CANCER_ERBB2_UP | 151 | 11 | 5.85E-5 |
| KEGG_PENTOSE_AND_GLUCURONATE_INTERCONVERSIONS | 28 | 6 | 5.85E-5 |
| GRAESSMANN_APOPTOSIS_BY_DOXORUBICIN_DN | 1757 | 40 | 5.85E-5 |
| REACTOME_INNATE_IMMUNE_SYSTEM | 1113 | 30 | 6.69E-5 |
| FLECHNER_BIOPSY_KIDNEY_TRANSPLANT_REJECTED_VS_OK_DN | 553 | 20 | 8.42E-5 |
| CUI_TCF21_TARGETS_2_DN | 850 | 25 | 1.33E-4 |
| BENPORATH_NANOG_TARGETS | 988 | 27 | 1.8E-4 |
| ONDER_CDH1_TARGETS_1_UP | 139 | 10 | 1.8E-4 |
| KRIGE_RESPONSE_TO_TOSEDOSTAT_6HR_UP | 931 | 26 | 1.81E-4 |
