# Supplementary material for: Hepatitis B virus compartmentalization and single-cell differentiation in hepatocellular carcinoma
Source: Life Sci Alliance. 2021 Jul 21;4(9):e202101036. doi: 10.26508/lsa.202101036 (PMC8321681; doi:10.26508/lsa.202101036)
Supplement: Supplementary file 7 [file LSA-2021-01036_TableS7.docx]

**Supplementary Table 7.** Tumor suppressor genes (TSG) highly negatively correlated (Rho< -40) with HBV-RNA in single cells. No oncogene was found to be highly positively correlated (Rho>40) with HBV-RNA. Data from Cancer Gene Census (CGC) database (<https://cancer.sanger.ac.uk/census>).

| **Gene** | **Tumor Type** | **Role in Cancer** | **Rho** | **p-value** |
| --- | --- | --- | --- | --- |
| ARHGAP26 | AML, MDS | TSG, fusion | -0.73581 | 0.000739 |
| GATA1 | megakaryoblastic leukaemia of Downs syndrome | oncogene, TSG | -0.6993 | 0.014539 |
| PPARG | follicular thyroid | TSG, fusion | -0.66176 | 0.006541 |
| POLD1 | CRC | TSG | -0.63377 | 0.002554 |
| RB1 | retinoblastoma, sarcoma, breast, small cell lung carcinoma | TSG | -0.6289 | 0.00045 |
| TET1 | AML | oncogene, TSG, fusion | -0.6262 | 0.002278 |
| CHEK2 |  | TSG | -0.62088 | 0.026867 |
| FAT1 | oral squamous cell, chemorefractory CLL, head and neck, pancreatic acinar cell carcinoma | TSG | -0.5933 | 2.39E-06 |
| NRG1 | NSCLC | TSG, fusion | -0.57982 | 1.22E-05 |
| FAT4 | lymphoma, pancreatic, head and neck, melanoma, hepatocellular carcinoma | TSG | -0.57276 | 0.01455 |
| STAG1 | colorectal cancer, AML | TSG | -0.57198 | 0.001769 |
| RUNX1 | AML, pre B-ALL, T-ALL | oncogene, TSG, fusion | -0.57087 | 0.000432 |
| TRIM24 | APL | oncogene, TSG, fusion | -0.53936 | 0.00069 |
| PMS2 |  | TSG | -0.53859 | 0.00356 |
| BAZ1A | uterine carcinosarcoma, colon cancer | TSG | -0.53059 | 0.002933 |
| NF1 | neurofibroma, glioma | TSG, fusion | -0.52308 | 0.000104 |
| DAXX | pancreatic neuroendocrine tumour, paediatric glioblastoma | oncogene, TSG | -0.51 | 0.010059 |
| MUTYH |  | TSG | -0.48772 | 0.035849 |
| FUS | liposarcoma, AML, Ewing sarcoma, angiomatoid fibrous histiocytoma, fibromyxoid sarcoma | TSG, fusion | -0.47992 | 0.001281 |
| TRAF7 | meningioma | TSG | -0.47955 | 0.002248 |
| LATS2 | malignant mesothelioma | TSG | -0.47385 | 0.017743 |
| NCOR2 | prostate | TSG | -0.4716 | 0.001389 |
| TSC1 | renal cell carcinoma, bladder carcinoma | TSG | -0.47005 | 0.001447 |
| RFWD3 | lung cancer | TSG | -0.46872 | 0.016678 |
| TNFRSF14 | follicular lymphoma | TSG | -0.46126 | 0.001239 |
| FBXO11 | DLBCL | TSG | -0.45858 | 0.004129 |
| TNFAIP3 | marginal zone B-cell lymphomas, Hodgkin lymphoma, PMBL | TSG | -0.45649 | 0.015464 |
| CLTCL1 | ALCL | TSG, fusion | -0.4384 | 0.001277 |
| ATR | endometrial, gastric, epithelial ovarian, myeloma | TSG | -0.43295 | 0.008871 |
| BAP1 | uveal melanoma, breast, NSCLC, RCC | TSG | -0.43265 | 0.004059 |
| SMARCB1 | malignant rhabdoid | TSG | -0.42962 | 0.014834 |
| FES | HNSCC, ovarian carcinoma | oncogene, TSG | -0.42956 | 0.020901 |
| EP300 | colorectal, breast, pancreatic, AML, ALL, DLBCL | TSG, fusion | -0.42824 | 0.001689 |
| SUZ12 | endometrial stromal tumour | oncogene, TSG, fusion | -0.41598 | 0.003522 |
| ARID2 | hepatocellular carcinoma | TSG | -0.41578 | 0.016805 |
| CHD2 | melanoma, large intestine, CLL, monoclonal B lymphocytosis | TSG | -0.40847 | 0.003822 |
| MAX | pheochromocytoma, endometrioid carcinoma, colon carcinoma | TSG | -0.40718 | 0.007857 |
| FOXO1 | alveolar rhabdomyosarcoma | oncogene, TSG, fusion | -0.4035 | 0.004743 |
| LARP4B | CRC, glioma | TSG | -0.40127 | 0.001942 |
| FANCA |  | TSG | -0.40077 | 0.048102 |
| SETD2 | clear cell renal carcinoma | TSG | -0.40009 | 0.003868 |

|  |
| --- |
